# Prophylactic Delivery of a Bacteriophage Cocktail in Feed Significantly Reduces Salmonella Colonization in Pigs

**DOI:** 10.1128/spectrum.00422-22

**Published:** 2022-05-17

**Authors:** Anisha M. Thanki, Guillaume Mignard, Robert J. Atterbury, Paul Barrow, Andrew D. Millard, Martha R. J. Clokie

**Affiliations:** a Department of Genetics and Genome Biology, College of Life Sciences, University of Leicester, Leicester, United Kingdom; b School of Veterinary Medicine and Science, University of Nottingham, Sutton Bonington, Leicestershire, United Kingdom; c School of Veterinary Medicine, University of Surrey, Daphne Jackson Road, Guildford, United Kingdom; University of California, San Diego

**Keywords:** *Salmonella*, bacteriophage therapy, bacteriophages, food-borne pathogens

## Abstract

Nontyphoidal Salmonella spp. are a leading cause of human food poisoning and can be transmitted to humans via consuming contaminated pork. To reduce Salmonella spread to the human food chain, bacteriophage (phage) therapy could be used to reduce bacteria from animals’ preslaughter. We aimed to determine if adding a two-phage cocktail to feed reduces Salmonella colonization in piglets. This first required spray drying phages to allow them to be added as a powder to feed, and phages were spray dried in different excipients to establish maximum recovery. Although laboratory phage yields were not maintained during scale up in a commercial spray dryer (titers fell from 3 × 10^8^ to 2.4 × 10^6^ PFU/g respectively), the phage titers were high enough to progress. Spray dried phages survived mixing and pelleting in a commercial feed mill, and sustained no further loss in titer when stored at 4°C or barn conditions over 6 months. Salmonella-challenged piglets that were prophylactically fed the phage-feed diet had significantly reduced Salmonella colonization in different gut compartments (*P* < 0.01). 16S rRNA gene sequencing of fecal and gut samples showed phages did not negatively impact microbial communities as they were similar between healthy control piglets and those treated with phage. Our study shows delivering dried phages via feed effectively reduces Salmonella colonization in pigs.

**IMPORTANCE** Infections caused by Salmonella spp. cause 93.8 million cases of human food poisoning worldwide, each year of which 11.7% are due to consumption of contaminated pork products. An increasing number of swine infections are caused by multidrug-resistant (MDR) Salmonella strains, many of which have entered, and continue to enter the human food chain. Antibiotics are losing their efficacy against these MDR strains, and thus antimicrobial alternatives are needed. Phages could be developed as an alternative approach, but research is required to determine the optimal method to deliver phages to pigs and to determine if phage treatment is effective at reducing Salmonella colonization in pigs. The results presented in this study address these two aspects of phage development and show that phages delivered via feed prophylactically to pigs reduces Salmonella colonization in challenged pigs.

## INTRODUCTION

Nontyphoidal Salmonella species are responsible for 93.8 million infections annually and 155,000 related deaths worldwide, of which 85% of human infections are foodborne (1, 2). In the EU, 10–20% and in the U.S. 9–15% of human salmonellosis infections were linked to consumption of contaminated pork meat (3–6). In pigs, Salmonella is spread by the fecal-oral route, and pigs can become infected at any stage of the farm to fork process (7, 8). As infections in pigs are enteric, the intestinal contents become infected, which increases the probability of carcass contamination and the presence of Salmonella in the human food chain (5). Consequently, controlling the spread of Salmonella preslaughter is a potential control point as it would reduce the introduction of the bacterium into the food chain and reduce risk to consumers.

Antibiotics are frequently used to treat infections in pigs, but broad-spectrum antibiotics are increasingly seen as being problematic in terms of driving antimicrobial resistance (9). In addition, bacterial 16S rRNA gene profiling studies have shown antibiotics affect the microbial populations in pig intestines, and subsequently cause conditions that favor growth of pathogenic E. coli (10, 11). Furthermore, antibiotics are losing their effectiveness as evidenced by an increasing number of strains becoming resistant to front-line antibiotics (12, 13). Consequently, alternatives to antibiotics are needed and bacteriophages (phages) could provide an effective solution. Phages are viruses that specifically infect and kill bacteria and are usually restricted to a genus or species. Studies have shown lytic phages can lyse multidrug-resistant (MDR) strains, such as representative strains from bacterial species P. aeruginosa (14), E. coli (15) and Salmonella strains isolated from pigs and chickens (16–18).

There is increasing interest in investigating therapeutic applications of phages in livestock (19–21). In these infection model studies, phage delivery methods such as via oral gavage, adding phages to drinking water and feed, have been tested. Delivering phages via oral gavage was tested by Wall et al. (2010), in which challenged pigs were administered a phage cocktail every 2 h over 6 h. Although phage treatment reduced Salmonella numbers in cecal samples by 1.4 log_10_ CFU/mL in comparison to the challenge control (22), delivering phages by oral gavage is technically challenging. It would not be practically possible for farmers to treat multiple individual infected pigs using this method. Instead, a delivery method is needed where multiple animals can be treated at the same time, such as in feed or in water. Borie et al. (2008) tested the delivery of a three-phage cocktail in drinking water, and treatment significantly reduced intestinal Salmonella intestinal colonization (*P* < 0.01) (23).

Phage delivery via water would be convenient but could limit product shelf life, and in addition large volumes of liquid phages are not easily transported (24). Concentrated, high titer phage stocks could be added to drinking water as either a concentrate or a powder, but again there is room for user error (20). Feed-based delivery is an alternative solution with many benefits, and one option to produce feed with phage is to mix dried phages with feed before pelleting. Recent advances in phage formulation work at laboratory scale have shown phages can be lyophilized, freeze-dried, or spray dried to a fine powder using various excipients (25, 26). Excipients protect phages against dehydration and act as water replacing agents to stabilize the virion particle during drying (27). Common phage drying excipients include the sugars trehalose, sucrose, and mannitol; the amino acid leucine; and the commercially available food grade pH responsive polymer Eudragit S100 (28, 29), all of which have been approved by the Food and Drug Administration (FDA).

The dried phage preparations are protected from thermal stresses due to the addition of excipients, which helps to improve their shelf lives, and studies have reported dried phages have minimal phage loss when stored at room temperature for 6 to 12 months, which would remove the need for cold storage (30, 31). Furthermore, a study compared long-term storage of Staphylococcus phages and showed phages stored at −20°C in excipients trehalose, sucrose, and glycerol had comparable stability. In comparison, liquid phages stored in phage suspension buffer had varying stability when stored at 4°C and at room temperature (32).

To date, there are no published studies on optimizing phage drying at commercial scale, which is clearly needed to develop a commercial product to add to feed. However, commercial companies have filed patents based on phage drying, which suggests growing interests in developing dried phage products. Companies include Omnilytics (patent number WO2009046138A1), Phagelux (WO2020254967A1), Ecolab (US20170064972A1), and Deerland, who are developing the probiotic phage product PreforPro (US9839657B2) (33), although their drying protocols have not been publicly shared. In addition to spray drying phages, data is required about the viability of phage when mixed with feed and whether they can survive the pelleting process used in pig feed production. In the standard feed manufacturing process, vitamins, minerals, and metals (premix) are added before pelleting. Therefore, adding phages at this stage of production would be ideal and would not add extra steps postpelleting.

This study was designed to address knowledge gaps for both the ability of phages to survive the milling process and whether dried phages mixed with feed could reduce Salmonella colonization in pigs. For both questions, we used a two-phage cocktail, consisting of myoviruses SPFM10 and SPFM14, as they had excellent host-specificity and can infect multidrug-resistant strains representative of the most common serotypes associated with pigs, which are *S. Typhimurium*; *S*. 4,12:i:-; *S*. 4,5,12:i:-; *S. Bovismorbificans*, and S. Derby (16). Phage SPFM10 was previously isolated from samples collected from a food processing plant on an *S. Typhimurium* strain, SPFM14 was isolated from pig feces on an *S*. 4,12:i:- strain, and both phages share the same propagation host (16, 18). To add the phage cocktail to feed, the phages were dried in order to limit moisture levels in the feed. We first optimized the phage drying process using a laboratory scale spray dryer and then scaled up the production by using a commercial spray dryer. Dried phages mixed with feed survived the pelleting process and remained stable in feed for over 6 months. Salmonella challenged piglets were fed the phage treated feed diet, and the addition of phages was able to reduce Salmonella colonization in different gut compartments in piglets and did not negatively affect their microbiome. Our study showcases data on the first steps in developing a commercial dried phage product to reduce Salmonella colonization in pigs.

## RESULTS

### Spray drying phages at laboratory scale.

**FIG 1:**
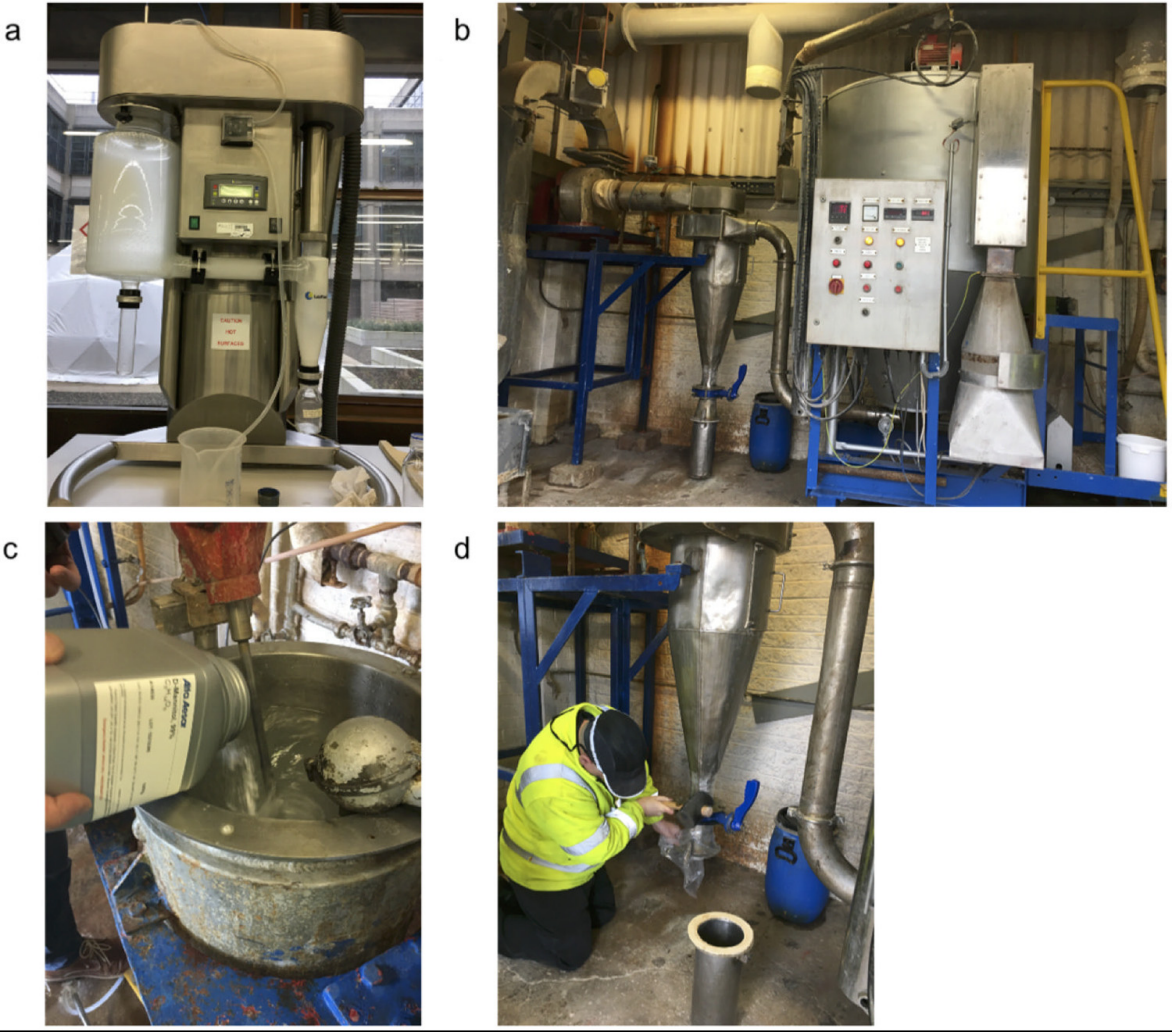
Images of the equipment used for spray drying phages. (a) Laboratory scale spray dryer used to dry phages SPFM10 and SPFM14. (b) Industrial spray dryer used to dry the phages that were then added to the weaner meal and pelleted. To dry the phages in the industrial scale dryer, the excipients, phages and water were mixed together in vessel (c) and dried powder was collected in tube (d).

**FIG 2:**
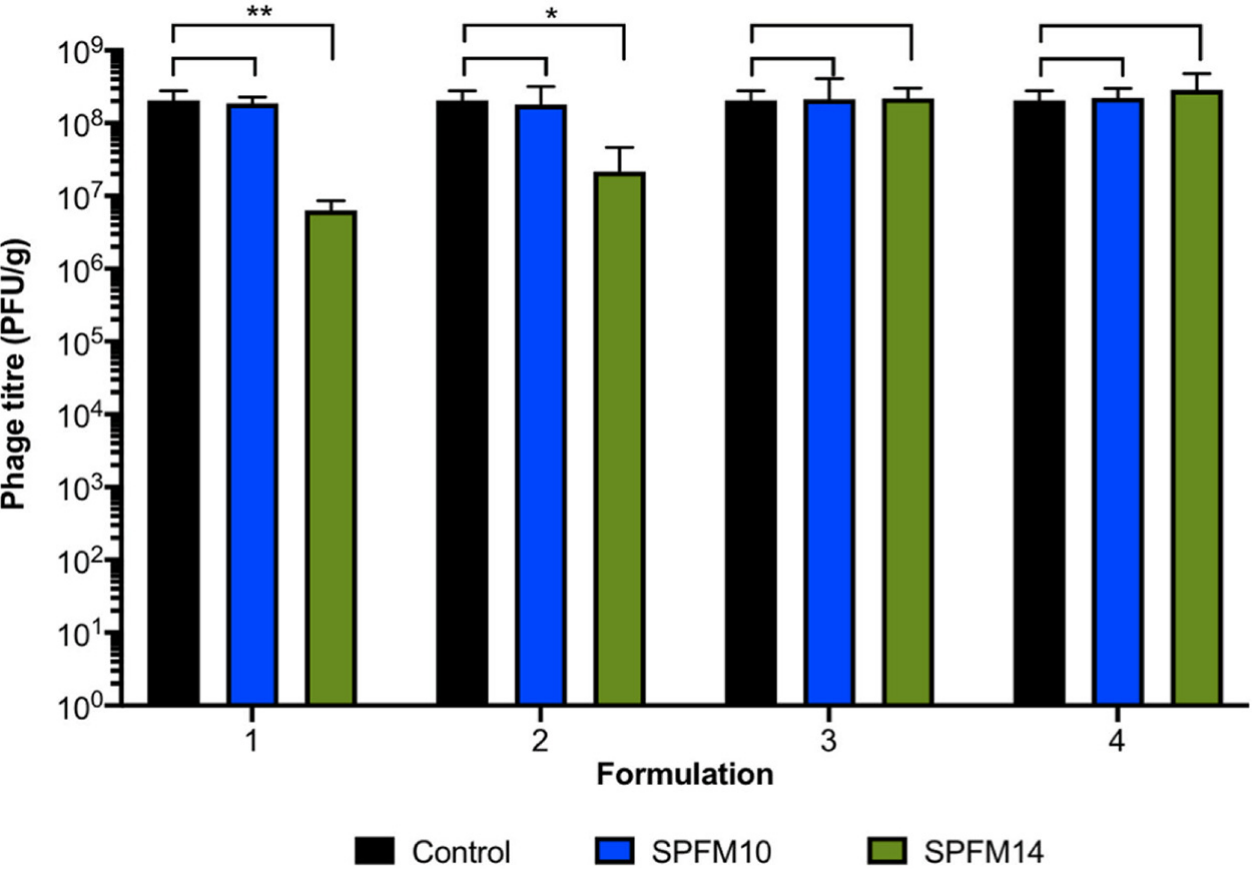
Testing stability of phages during spray drying. Phages SPFM10 (blue bars) and SPFM14 (green bars) were sprayed dried with different excipients, which were 4% trehalose (formulation 1); 4% trehalose, 4% mannitol (formulation 2); 4% trehalose, 4% mannitol, and 1% leucine (formulation 3); 4% trehalose, 4% mannitol, 1% leucine, and 2% polymer Euradagit (formulation 4). Differences in phage titers following drying were compared to the input titer (black bars), and statistical differences are displayed on the graph (*, *P* < 0.05; **, *P* < 0.01). Data presented are average titers from three biological replicates, each with three technical repeats, and error bars represent the standard error of the mean (SEM).

Phages SPFM10 and SPFM14 were spray dried with different excipient formulations using a laboratory scale spray dryer (Fig. 1a). The same inlet and corresponding outlet temperatures were used in all cases to allow comparison of all formulations, and to determine which formulation protected the phages most effectively. The following were tested: formulation 1 was 4% trehalose; formulation 2 was 4% trehalose and 4% mannitol; formulation 3 was 4% trehalose, 4% mannitol, and 1% leucine; 4% mannitol; formulation 4 was 4% trehalose, 4% mannitol, 1% leucine, and Euradagit S100 (Fig. 2). SPFM10 was stable during spray drying. and there were no significant reductions in phage titer across all four formulations (*P* > 0.05). In comparison, there were significant reductions in phage titer for phage SPFM14 when dried with formulations 1 and 2 (*P* = 0.04). SPFM14 was more stable when dried with formulations 3 and 4 (*P* > 0.05).

### Spray drying phages at commercial scale.

To scale up production of dried phages, a commercial scale spray dryer was used (Fig. 1b to d). However, opposite to laboratory dryers, the temperatures used for the commercial dryer are controlled by the outlet temperature rather than the inlet. Consequently, the process required significant optimization of different outlet and inlet temperature combinations and excipient formulations. The impact of all conditions and formulations on phage titer are listed in Table S1 in the supplemental material and are labeled as runs A–T.

From the optimization study it was concluded that the formulation 8% trehalose, 8% mannitol, 1% leucine, and 2% Euradagit S100 mixed with 50% phage (50% of total volume) used in run S was most effective, as phage recovery was highest at titer 2.4 × 10^6^ PFU/g (Table S1). The run was with an outlet temperate of 60°C and inlet temperature of 110°C. The formulation and drying conditions of run S were used to spray dry SPFM10 and SPFM14, and 32.5 kg of each formulated phage was produced.

### Pelleting and stability of dried phages in feed.

To determine if dried phages could withstand conditions used during commercial feed pelleting, 65 kg of total phage powder at titer ~2.0 × 10^9^ PFU/kg was mixed with 1 ton of Provimi early weaner meal (Fig. S1 and Table S2). This large volume was required as 1 ton of feed was the minimum manufacturing capacity of the mill and it was of interest to test phage stability at commercial scale production. The average phage titer before pelleting with the Provimi early weaner meal was 1.30 × 10^5^ PFU/g and following pelleting was 9.5 × 10^4^ PFU/g; thus, there was no significant decrease (*P* = 0.1)

**FIG 3:**
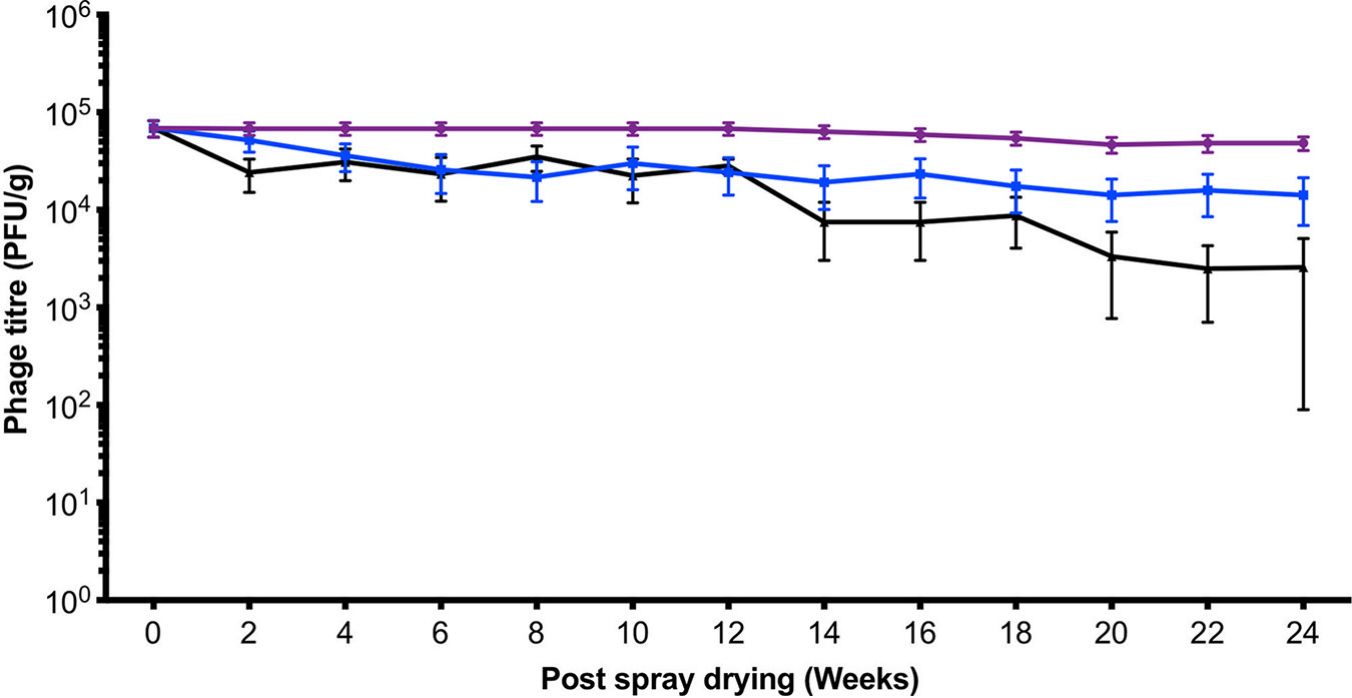
Stability of dried phages in feed. Spray dried phages were mixed with the weaner meal and pelleted using Cargill’s commercial mill. The stability of phage in feed stored at three different conditions at 4°C (purple), in a barn at Harper Adam’s University (blue), and at −20°C (black) was monitored over 24 weeks. Data presented are average titers from 12 biological replicates, each with three technical repeats.

After pelleting, the feed containing the phage preparation was stored at three different conditions: 4°C, in a barn (where temperatures ranged from 10 to 25°C during storage), and at –20°C to determine how storage temperature impacts phage stability within the feed (Fig. 3). The phage titer for feed stored at 4°C remained stable over 6 months and on average when stored in a barn fell from 9.5 × 10^4^ to 1.42 × 10^4^ PFU/g after 6 months storage (*P* = 0.04). The greatest reduction was observed when the phage-in-feed was stored at –20°C, where average counts decreased from 9.5 × 10^4^ to 2.58 × 10^3^ PFU/g after 6 months (*P* < 0.001). Furthermore, in 6/12 of the 10 g feed samples screened, no phages were detected, which highlights that –20°C conditions are not ideal for long-term phage in feed storage.

### Challenge piglet study.

For the study, animals were divided into four groups: the control group (T1); piglets fed the phage treated diet only (T2); piglets challenged with Salmonella (T3); and piglets challenged with Salmonella and fed the phage treated diet (T4). Four days after the piglets arrived on the trial site, one piglet from T1 was lame and given antibiotics. This piglet was excluded from the study, and for the remaining part of the study there were only two piglets in T1.

### Fecal shedding of Salmonella and phages.

**FIG 4:**
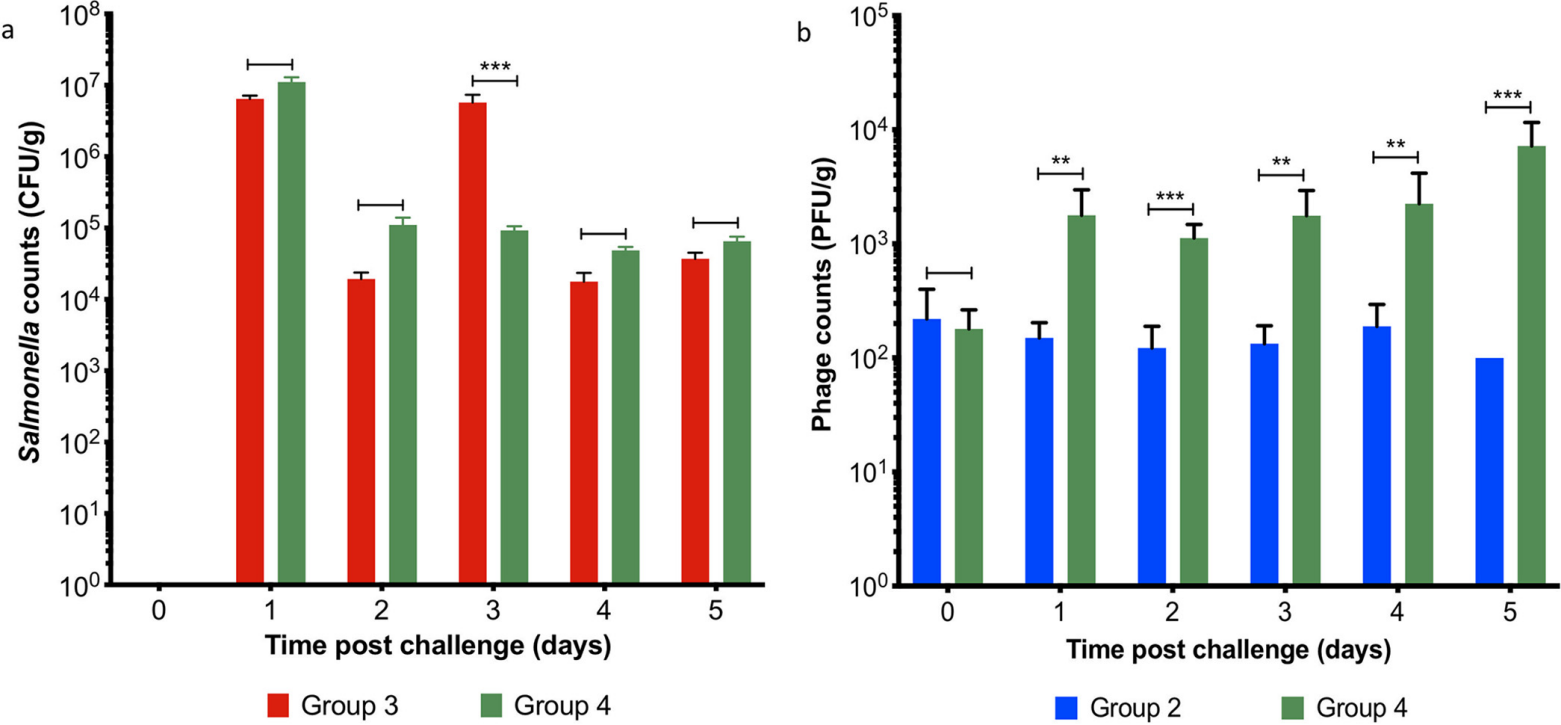
Pig fecal shedding of Salmonella and phages during the phage therapy trial between treatment groups. Between study days 0 to 5, Salmonella shedding (a) was monitored daily for pigs in groups: T3 (Salmonella challenged—red bars) and T4 (Salmonella challenged and fed the phage-feed diet—green bars). Simultaneously, fecal phage shedding (b) was enumerated, for pigs in groups T2 (fed the phage-feed diet—blue bars) and T4 (green bars). Data presented are averages in each treatment group and statistical differences between treatment groups are displayed on the graph (**, *P* < 0.01; ***, *P* < 0.001).

All piglets tested negative for Salmonella when they arrived at the trial site, 3 days prior to challenge, and on challenge morning. Postchallenge on study day (SD) 1, Salmonella counts were on average 8.77 × 10^6^ PFU/g in collected fecal samples from challenged piglets in groups T3 and T4 (Fig. 4a). On SD 2, the average Salmonella counts in feces were not significantly different between T3 and T4 (*P* = 0.0518). However, on SD 3 fecal shedding of Salmonella was on average 9.27 × 10^4^ CFU/g in piglets fed the phage treated diet in group T4, which was significantly less compared to Salmonella shedding in T3 (*P* < 0.001). Despite this reduction in fecal shedding, on SDs 4 and 5 fecal Salmonella shedding in T3 and T4 were similar. As expected, Salmonella was not isolated from fecal samples collected from unchallenged piglets in groups T1 or T2.

A particularly interesting comparison is the amount of fecal shedding of phages that was detected from piglets fed the phage treated diet in group T2 and in T4 where piglets were challenged with Salmonella and fed the phage treated diet (Fig. 4b). On SD 0, the phage counts were similar between the groups (on average 2.00 × 10^2^ PFU/g). However, from SDs 1 to 5, phage counts from group T4 were consistently on average approximately 10 times higher in the infected animals compared with group T2, which suggests there is phage replication postchallenge. Phages were not detected in fecal samples collected from piglets in groups T1 and T3, which were not fed the phage treated diet.

### General health of piglets throughout study.

The health of piglets was monitored daily postchallenge. The rectal temperatures of piglets in T1 and T2 remained constant over the study days at ~38.3°C. In comparison, the temperature of piglets in T3 and T4 increased on SD 1 to 40.3°C from ~38°C on SD 0, but on SD 5 their temperatures were ~38.8°C (Table S3). The rise in temperature is likely linked to Salmonella infection. The piglets in T1 and T2 gained weight over the course of the trial by 1 kg and 1.48 kg, respectively. In comparison, the weight gain of piglets in groups T3 and T4 were 0.69 kg and 0.19 kg, respectively (Table S4). Group T3 had higher weight gain than T4 (*P* < 0.05).

Piglets in groups T3 and T4 experienced diarrhea consistent with Salmonella infection from SDs 2 to 5 in groups T3 and T4. In groups T1 and T2, fecal samples were observed to be normal and firm (Table S5). There were no signs of depression, respiratory distress, or coughing in all piglets across the treatment groups. The daily feed intake from SD -3, after the treated diet was introduced to SD 5, was comparable for piglets in group T1 and T2. The feed intake for piglets in groups T3 and T4 after challenge reduced by half in comparison to T1 and T2 (Table S6).

### Salmonella and associate phage counts post sacrificing of piglets.

**FIG 5:**
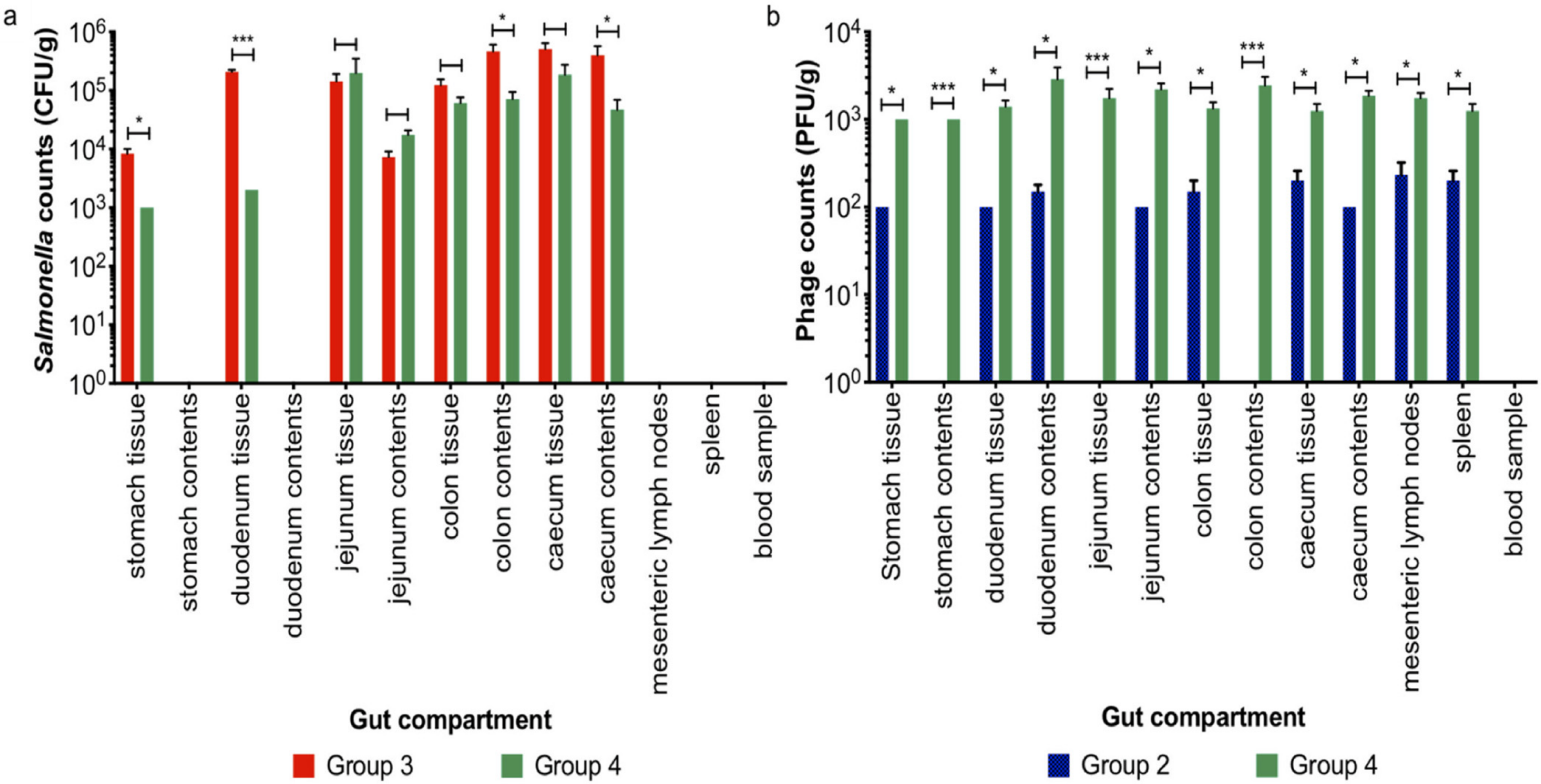
Salmonella and phage counts in different gut compartments in the pigs. Graph (a) shows isolated Salmonella numbers from pig gut and blood samples from groups T3 (challenged with Salmonella—red bars) and T4 (challenged with Salmonella and fed the phage-feed diet—green bars). (b) Phage numbers recovered from pig guts and blood samples collected from groups T2 (fed the phage-feed diet—blue bars) and T4 (green bars). Data presented are averages in each treatment group and statistical differences between treatment groups are displayed on the graph (*, *P* < 0.05; ***, *P* < 0.001).

Gut compartment samples, including tissue and contents samples, were collected on SD 5, and they showed there were significant differences in Salmonella colonization between groups T3 (challenged with Salmonella) and T4 (challenged with Salmonella and fed the phage treated diet) (Fig. 5a). In the stomach tissue, Salmonella counts in group T4 were reduced by on average 1.00 × 10^1^ CFU/g (*P* < 0.05), in the duodenum tissue by 1.05 × 10^2^ CFU/g (*P* < 0.001), in the colon contents by 1.00 × 10^1^ CFU/g (*P* < 0.05), and in cecum contents by 1.00 × 10^1^ (*P* < 0.05) in comparison to the gut samples from group T3. Thus, suggesting phage treatment reduced colonization.

Salmonella counts on average were below the limit of detection (100 CFU/g) in samples from stomach contents, duodenum contents, mesenteric lymph node, spleen, and blood samples from both groups T3 and T4. In contrast, phages were isolated in all gut compartment samples collected from T4 (Fig. 5b). This indicates phages were transiting through the gut, which could explain why phages were isolated in sites where Salmonella was not isolated. In all gut compartments collected from T4 piglets, average phage counts were 10-fold higher than the similar samples collected from T2 (unchallenged and fed phage treated diet) (*P* < 0.05). The higher phage counts recovered in T4 samples suggests phages were actively replicating due to challenge. In T2 gut samples, phages were not isolated from the stomach, jejunum tissue, or colon contents. In comparison, in the same samples collected from group T4, the average phage counts were ~2 × 10^3^ PFU/g (*P* < 0.001), which again highlights phages were transiting though the gut and colonizing. In both groups T2 and T4 no phages were detected in the blood samples.

### 16S *rRNA* gene sequencing.

16S rRNA gene sequencing was conducted to determine if there were any differences in microbial communities in samples of feces, cecum, and colon contents between the treatment groups over the course of the trial. Sequencing was conducted on fecal samples collected on SD 0 and on SDs 3 and 5 postchallenge, and on cecum and colon contents samples collected on SD 5. The cecum and colon samples were selected for study as these are the gut compartments where Salmonella colonizes heavily in pigs.

### Alpha and beta diversity between samples and treatment groups.

**FIG 6:**
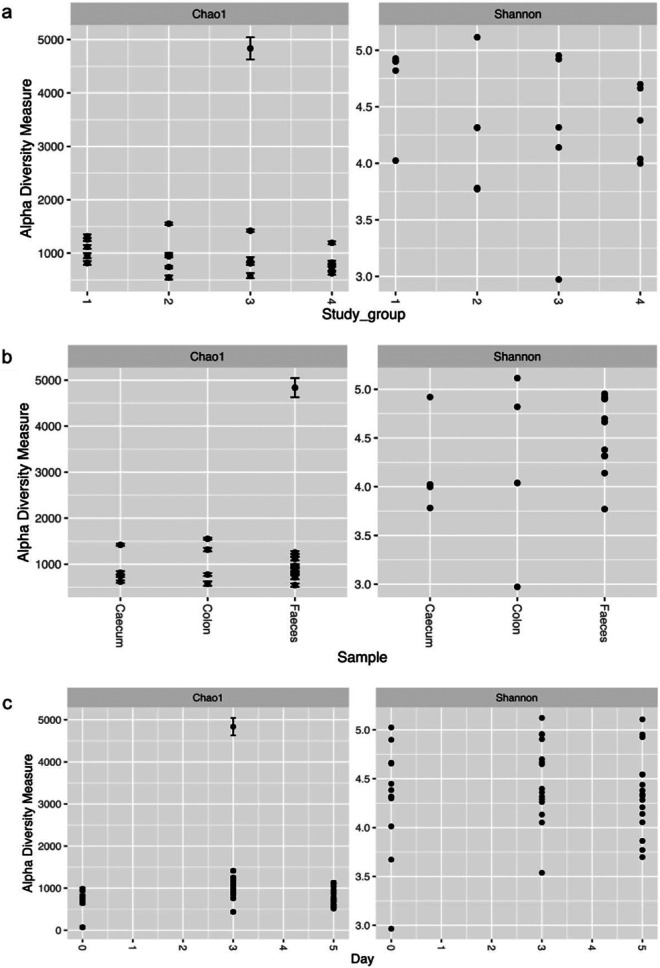
The graphs show alpha diversity between samples in different treatment groups (a), between samples (fecal, cecal, and colon) (b), and between study days (0–5) (c). The Chao1 and Shannon indexes were used to measure alpha diversity.

**FIG 7:**
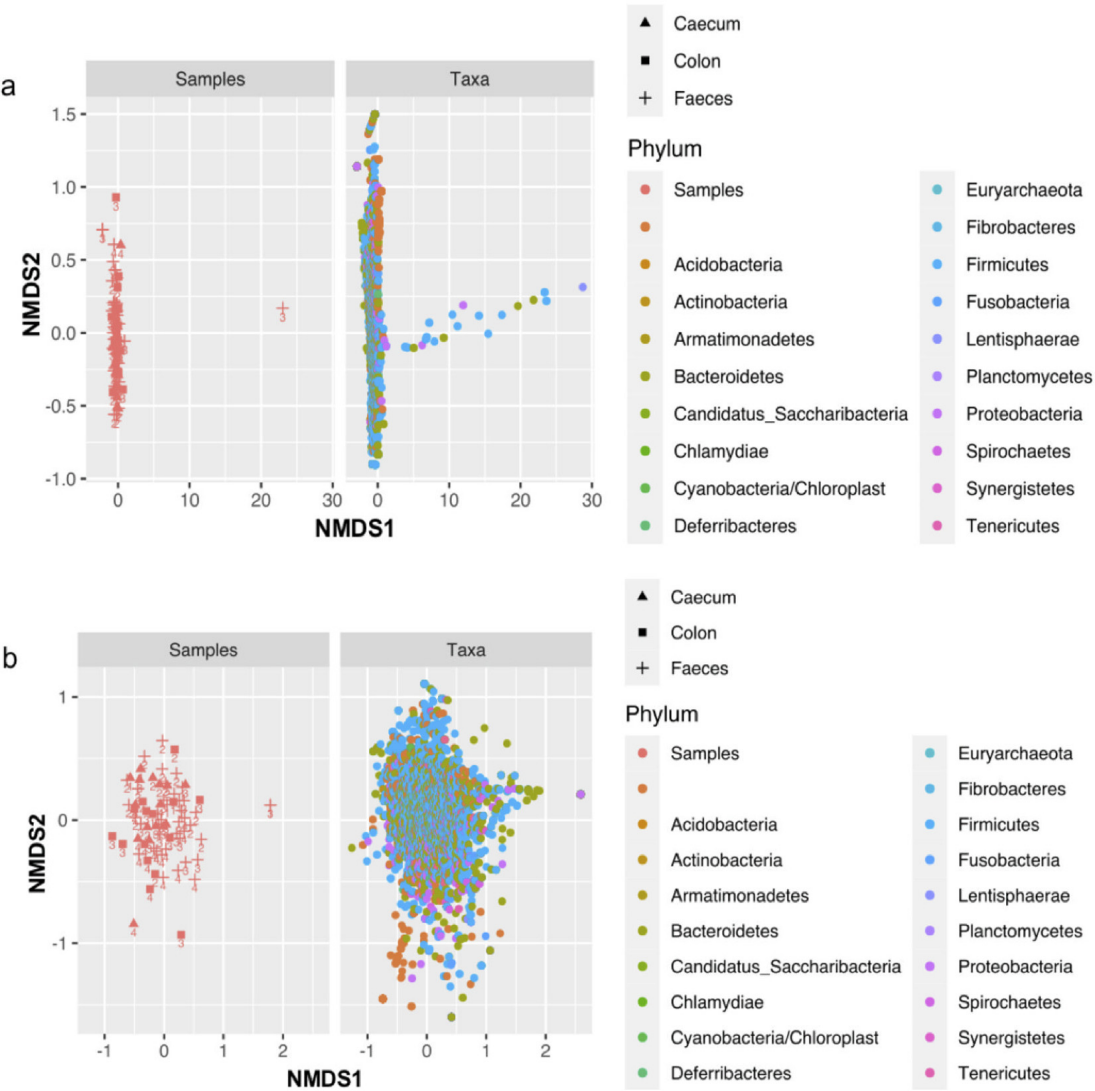
The NMDS plots show beta diversity of samples and taxa collected from different treatment groups (a). An outlier was identified in a fecal sample collected from T3 and was removed to determine diversity of the remaining samples (b). The colored points refer to samples (cecum, colon, and fecal) collected from different treatment groups the numbers on the point.

Comparison of alpha diversity metrics (Shannon and Chao1) indicated there was no significant difference (Mann-Whitney *P* > 0.05) between the microbial compositions of samples in different treatment groups; between the fecal, cecum, and colon samples; and between fecal samples collected on different study days (Fig. 6), although the Chao1 index highlighted there was one outlier in T3, which was a fecal sample collected on day 3. The beta diversity results also highlighted the outlier on the NMDS plot (Fig. 7a), but when the outlier was removed, all the fecal, cecum, and colon samples from different treatment groups clustered together, suggesting their microbial communities are similar (Fig. 7b). The outlier observed in T3 is linked to a piglet who became very sick following the Salmonella challenge, which could be a possible explanation to the difference in the microbial communities observed.

Further beta analysis was conducted to determine if there were differences in microbial communities for other variables used in the study. The variables were as follows: differences between samples in different study group; different sample types; samples taken from challenged or unchallenged pigs; and samples taken from piglets fed the treated phage diet and the untreated diet (Fig. S2). Permutational multivariate analysis of variance (PERMANOVA) statistical analyses showed there was only a significant difference between microbial communities in samples taken from pigs fed the treated phage diet or the untreated diet (*P* = 0.039).

### Evaluation of the taxonomic composition between the pig treatment groups.

**FIG 8:**
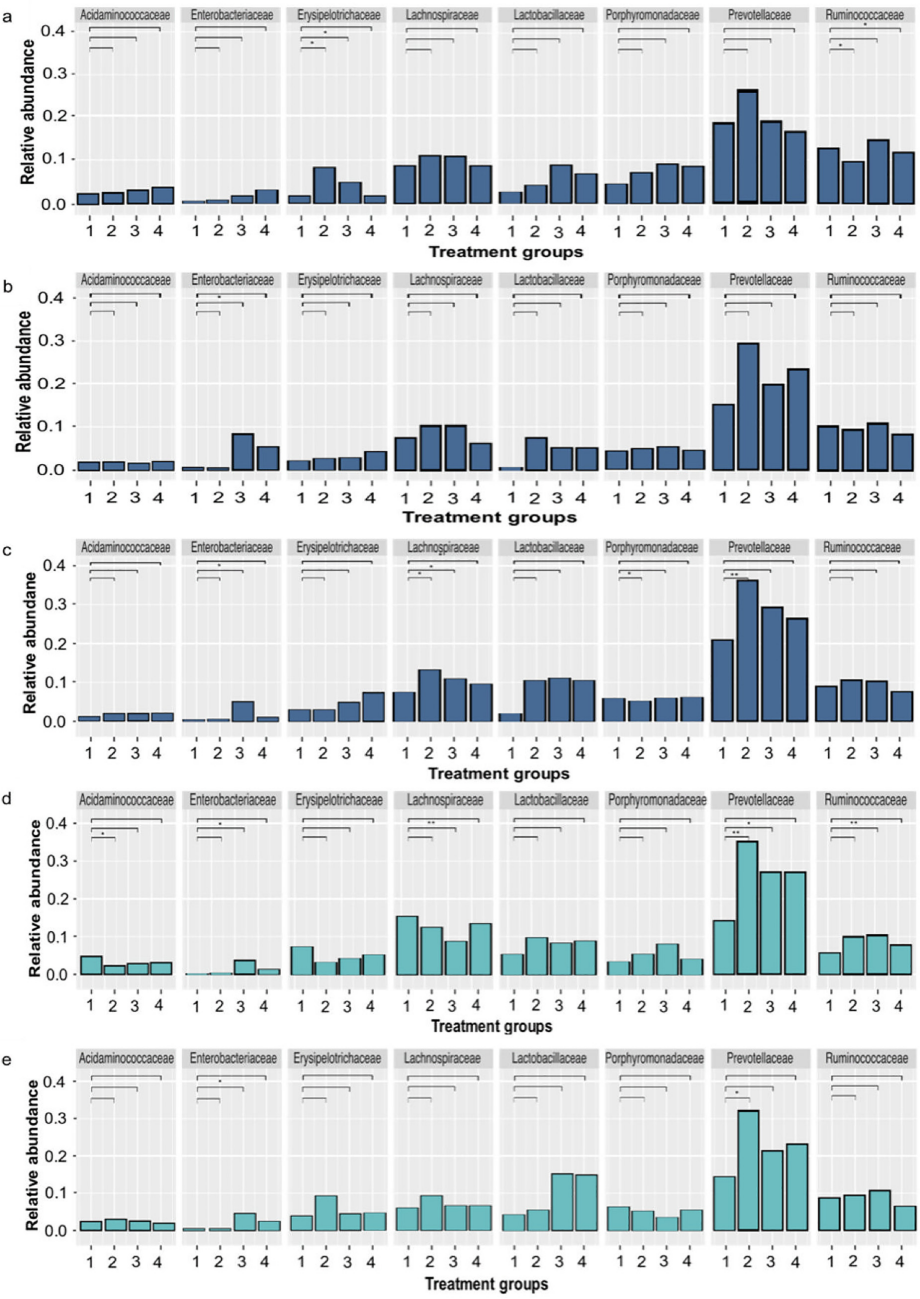
Relative abundance of microbial communities in fecal, cecal, and colon samples from pigs at the family level. The pig fecal samples were collected 0 (a), 3 (b), and 5 (c) days postchallenge with Salmonella from treatment groups T1 (control), T2 (fed the phage-feed diet), T3 (challenged with Salmonella), and T4 (challenged with Salmonella and fed the phage-feed diet) and are shown in the dark blue bars. The cecum (d) and colon (e) samples were collected 5 days postchallenge from all treatment groups during the postmortem (turquoise-colored bars). Each bar represents the mean relative abundance of the treatment group, and relative abundance of the top 8 families are displayed. The relative abundance of each family in T1 (labeled as 1 on the graph) samples was compared to samples collected from groups T2 (2), T3 (3), and T4 (4). Statistical differences between treatment groups are displayed on the graph (*, *P* < 0.05; **, *P* < 0.01).

The relative abundance of microbial communities at the family level were compared between the control pigs in T1 versus treatment groups T2 (unchallenged and fed the phage treated diet), T3 (challenged with Salmonella), and T4 (challenged with Salmonella and fed the phage treated diet) on SD 0 before challenge, and after challenge on SDs 3 and 5 (Fig. 8). Across all treatment groups and study days, Prevotellaceae was consistently the most abundant family found in the fecal bacterial microbiota.

In fecal samples collected on SD 0, there was no significant difference in the relative abundance of the Enterobacteriaceae family, which includes the genus Salmonella (Fig. 8a). However, on SDs 3 and 5 the relative abundance was significantly higher in T3 (*P* = 0.0108) in comparison to T1 (Fig. 8b and c). In comparison, the relative abundances of Enterobacteriaceae between T1 and T4 did not differ significantly (*P* = 0.3784) on SDs 3 and 5. Other changes on SD 5 were that the relative abundance of the Porphyromonadaceae was lower in T4 in comparison to T1 (*P* = 0.0464) and the relative abundance of Prevotellaceae was higher in T2 in comparison to T1 (*P* = 0.0059) (Fig. 8c). Prevotellaceae have been associated with healthy pigs (34), and thus possibly phage treatment had a positive effect on the microbiota.

In the cecum, the relative abundance of Acidaminococaceae was lower (*P* = 0.0059) and abundance of Prevotellaceae was higher (*P* = 0.0048) in T2 versus T1. The relative abundance of Lachnospiraceae (*P* = 0.0023), Prevotellaceae (*P* = 0.0143), and Ruminococcaceae (*P* = 0.048) were significantly different between T3 Salmonella challenged piglet samples versus T1 control piglets (Fig. 8d). In the colon, the relative abundance on the Enterobacteriaceae in T3 was higher (*P* = 0.0481) in comparison to T1, and the abundance of Prevotellaceae was higher (*P* = 0.0296) in T2 versus T1 (Fig. 8e). There were no significant differences in the relative abundance of the top families between T1 and T4 in both colon and cecum samples (*P* > 0.3), which indicates phages were not negatively affecting the microbiota.

## DISCUSSION

In this study, we show that dried phages can survive the animal feed pelleting process and therefore a stable phage feed product can be produced. We also show that when the treated phage-feed diet is fed to challenged piglets, the change in diet significantly reduces Salmonella colonization throughout the gut. The two phages selected for the study were previously characterized, and SPFM10 was found to be naturally heat-stable and surviving up to 80°C in temperature without loss in activity (16, 18). This heat stability could explain why the phage remained stable during drying using a laboratory scale spray dryer. In contrast, there were significant reductions in phage titer when the more heat liable phage SPFM14 was dried with different sugar excipients. Similar losses in phage titers have been observed for other phages dried with sugars and polymers (29, 35). SPFM14 stability was improved when sugar excipients were combined with an amino acid and polymer (formulations 3 and 4). The hydrophobic amino acid leucine has been shown to improve phage recovery as it protects against moisture during the drying process (28). Addition of the polymer to formulation 4 also improved yield, and combining the polymer with sugars and leucine excipients further helped to stabilize the phages during drying.

Our data illustrate that although spray drying phages at laboratory scale provides insights into phage stability, it can only guide optimal drying formulations. Translating the technology from the bench to commercial scale settings requires extensive optimization, and to our knowledge we are the first to show the scale-up data in a mainstream commercial spray dryer. The first hurdle we faced was that the parameters used for drying at commercial scale differed from the laboratory scale dryer as the outlet temperature dictated the inlet temperature of the commercial dryer, which is the opposite way around for a laboratory scale spray dryer. Furthermore, the higher temperatures used commercially, inactivated the phages. Significant phage recovery was only achieved after the outlet temperature was lowered to below 54°C and by testing different excipient formulations. Despite retrieving phages from the commercial dryer, there were significant losses in titer, and further optimization is needed to improve recovery. Other excipients, such as the sugar lactose (28) and methacrylate polymer solution (36), could be tested, both of which have both been shown to protect phages during drying.

Despite this phage loss, for our study the recovered phage titer was sufficient for downstream experiments. First, we determined if dried phages remained stable when mixed with meal and pelleted using a commercial mill. We showed that dried phages were indeed able to survive the pelleting process, in which they encountered high temperatures and pressure to mold the pellets. This was a pleasant finding as the process inactivates some antibiotics and enzymes, which instead have to be added postpelleting (37) and incurs an extra step in the manufacturing process. Phage stability during pelleting could have been due to added protection provided by the excipients coating the phages and may have contributed to phages remaining viable when stored over 6 months.

Prophylactic administration of dried phages in feed were able to significantly reduce Salmonella colonization in the stomach, duodenum, colon, and cecum. Reductions in the bacterial counts were likely to be due to phages being able to reach the sites of infection in different gut compartments as phages did multiply in challenged pigs in group T4. Similar reductions in Salmonella counts have been observed in other phage therapy pig challenge studies, but these studies used much higher phage doses. Wall et al. administered a liquid phage cocktail at a dose of 10^9^ PFU/mL via oral gavage to challenged pigs. The treatment was able to reduce Salmonella colonization in pig cecal and ileal samples by 5.94 × 10^2^ CFU/mL (22). Another study showed administration of a phage cocktail at dose 3 × 10^9^ PFU/mL, 24 and 48 h after challenge was able to reduce Salmonella counts in the cecum and feces by 1.6 × 10^1^ CFU/g after 96 h (38). Our study shows even a low phage dose was sufficient to reduce Salmonella colonization, but more research is needed to determine the optimal phage dose.

There have been two studies to date that have tested efficacy of dried phages (using laboratory scale spray dryers) in E. coli challenge models. Stanford et al. conducted a study where they encapsulated four phages and spray dried. Then they tested two different oral delivery methods, which were by adding dried phages at titer 10^10^ PFU/mL to a gelatin capsule and by mixing phage with feed to barely-silage mixture at titer 10^11^ PFU/mL. However, despite the high phage titers used, both treatments were unable to reduce shedding of E. coli over 10 weeks in cattle (36). Another study delivered one phage in feed by pulverizing the phage via spray drying (39). They tested two different doses of phage in feed at 10^6^ and 10^8^ PFU/g, and both reduced shedding of enterotoxigenic E. coli by 250 CFU/mL 4 days postinfection (39). Again, despite both studies using high phage doses, the treatments were unable to cause significant reductions in bacterial colonization unlike our study.

The results from our study showed that administering phages in feed was safe as there were no negative side effects in piglets just fed the phage in feed (group T2) and their health was comparable to the control piglets based on parameters such as rectal temperatures, weight gain, and feed intake. To probe further, we investigated if there were any differences in microbial communities between the groups in fecal, colon, and cecum samples. Overall, the microbial communities were very similar for groups T1 and T2 for all samples, showing phages did not have a detectable negative impact on the intestinal microflora. Interestingly, the relative abundance of Prevotellaceae was consistently higher in all samples collected from group T2, which is the family that has been previously shown to be associated with healthy pigs (40, 41). Consistent with our study, another pig phage challenge study also observed that the relative abundance of the Prevotellaceae was higher in phage treated groups (42). Interestingly, the Prevotellaceae family has been experimentally shown to have positive associations with disease resilience and growth performances in pigs (43). In samples collected from Salmonella challenged piglets in group T3, the relative abundance of the family Enterobacteriaceae was significantly higher postinfection. The relative abundance was significantly lower in group T4 where piglets were challenged with Salmonella and fed the phage treated diet, which could suggest phages are effective at reducing Salmonella colonization in the different piglet gut compartments, and the data mirror the culture-based results.

In summary, our study provides insights on scaling up production of dried phages at commercial scale to produce a phage in feed product. We also show delivering phages via feed is a viable option as the diet effectively reduced Salmonella colonization in different gut compartments of piglets. Future work is needed to test efficacy of different phage doses in challenge studies, improving phage recovery when dried at commercial scale and determining levels of phage resistance in pigs.

## MATERIALS AND METHODS

### Strains and phages used in this study.

The laboratory reference strain Salmonella enterica subsp. Enterica serovar Typhimurium SL1344 (accession number FQ312003) was used in this study. Salmonella was routinely grown on Xylose Lysine Deoxycholate (XLD) agar (Oxoid, UK) overnight at 37°C. To prepare liquid cultures, strains were inoculated in Luria broth (LB) broth (Melford Biolaboratories Ltd, UK) and grown overnight at 37°C at 100 rpm. Phages SPFM10 and SPFM14 were used (16, 18).

### Phage propagation and titration.

Phages were propagated by mixing exponentially growing liquid cultures of Salmonella SL1344 (10^7^ CFU/mL) in LB broth with 10^7^ PFU/mL of phages. Samples were incubated at 37°C with shaking (100 rpm) for 6 h, after which they were centrifuged at 4,200 × *g* for 15 min, the supernatant was filtered with 0.22 μm pore-size filters, and lysates were stored at 4°C. To determine phage titers, phage lysate was serially diluted 10-fold in phage suspension buffer (100 mM NaCl, 8 mM MgS0_4_.7H_2_O and 50 mM Tris-Cl) and plaque assays were conducted on 1% (wt/vol) LB agar plates with a lawn of SL1344 (44). Plates were incubated overnight at 37°C and phage titers were expressed as PFU/mL.

### Spray drying phages at laboratory scale.

For the spray drying procedure previously described, methods were used with slight modifications (29, 35). Initially, excipients and phages at titers of 5 × 10^10^ PFU/mL (1% phage added to final volume) were dissolved in ultrapure water to make up a 200 mL volume solution. The excipients tested were trehalose (Glentham Life Sciences Ltd, UK), leucine (Glentham Life Sciences Ltd, UK), mannitol (Fisher Scientific, UK) and Eudragit S100 (Evonik, Germany). Eudragit S100 was dissolved in ultrapurified water (5% wt/vol) by the addition of 4 M NaOH (Fisher Scientific, UK) drop by drop, until the solution turned clear, which indicated polymer dissolution (26).

The excipient-phage solution was spray dried using a laboratory scale LabPlant Spray Dryer (UK) with a two-fluid nozzle for atomization, and the nozzle had a diameter of 0.5 mm (Fig. 1a). Air speed of 4.3 ms^−1^ and liquid flow rate of 280 mlh^−1^ were used. The drying temperature was controlled by changing the inlet temperature from 80°C to 100°C, and the corresponding outlet temperatures varied from 40°C to 60°C. Dried powder phages were passed through the cyclone, collected in 100 mL glass bottles, and stored at 4°C until use. To determine phage titer, 0.10 g of dried powder phage was suspended in 900 μL phage suspension buffer, diluted 10-fold, and titered by plaque assays (Kropinski et al., 2009).

### Spray drying phages at commercial scale.

The Niro production minor spray dryer (GEA, UK) was used for drying. The dryer had pump pressure set at 20 Mpa and had a water evaporation capacity of 5–30 kg/h (Fig. 1b). The spray dryer had a rotary slotted disc atomizer, which uses a high-speed rotating disk to divide liquid into droplets, and the maximum atomizer setting was 50 Hz.

Phages and excipients were mixed with ultrapure water in a vessel that had a magnetic stirrer (Fig. 1c), before being pumped through the spray dryer. Dried material was collected from the cyclone (Fig. 1d). Unlike with the laboratory scale spray, the outlet temperature dictated the inlet temperature and the drying parameters needed to be optimized. A range of outlet temperatures from 50 to 70°C were tested, which resulted in inlet temperatures 100 to 160°C. For the optimization process, excipients and phages at different final volumes were tested and for each run 3 L solution of phage-excipient-water were used. Excipients tested were trehalose, leucine, mannitol, and Eudragit S100, and the different excipient formulations tested are listed in Table S1. Moisture content of dried material was determined by using the HG53 Halogen moisture analyzer (Mettler Toledo, UK) and measurements were taken in triplicate (Table S1).

### Production of pelleted phages in feed.

The production line used for pelleting was initially cleaned by flushing with 2 tons of wheatfeed. Dried phages (65 kg in total; 32.5 kg SPFM10 and 32.5 kg of SPFM14) were mixed with Provimi early weaner meal, which included Provimi early weaner premix at an inclusion rate of 4.10% to make 1 ton of mix (Fig. S1 and Table S2). The mixture was pelleted according to industry procedure, a pressing temperature of ~55°C was used to pellet feed, and pellets were bagged in 25 kg bags. The line was cleaned by flushing with 10 tons of wheatfeed with 2% ProHacid, which was able to inactivate phages.

The phage-in-feed pellets were stored at Harper Adams University, United Kingdom, at three different conditions: in a barn (where temperatures ranged from 10 to 25°C during storage), at 4°C, and at −20°C. Every 2 weeks, 50 g from four different bags were taken from each storage condition (12 samples in total) and posted to the University of Leicester. To determine phage titer, 10 g of pellets were crushed, mixed with 20 mL of phage suspension buffer, placed on the shaker for 1 h, and centrifuged at 4,200 × *g* for 10 min, and the supernatant was filtered using a 0.22 μm pore-size filters (Millipore, UK). The supernatant was diluted 10-fold and were used for plaque assays to determine phage titer (44).

### Challenge piglet study.

The trial protocol was presented and accepted by the Ethical Review Body and Drayton Animal Health Welfare and conducted in accordance with the Animals (Scientific Procedures) Act 1986. The study was carried out by Drayton Animal Health Ltd (UK). Eighteen male piglets (species Sus scrofa *domestica*) were used in the study, and on arrival piglets were weighed and received the creep feed supplied by the commercial farm. Fecal swabs were taken from all piglets on arrival and 3 days prior to challenge to check for presence of Salmonella (method described below).

Piglets were allocated to four groups: T1, the unchallenged and untreated group; T2, the unchallenged and treated with the phage in feed diet group; T3, the challenged and untreated group; and T4, challenged and treated group. There were three piglets in group T1, and five piglets were allocated to each group T2, T3, and T4. Treatment groups T2 and T4 were housed in one room and piglets T1 and T3 were housed in a separate room. Piglets in the same treatment group were housed in the same pen.

The treated phage diet was introduced three study days prior to challenge to piglets in groups T2 and T4. Groups T1 and T3 received the nonphage supplemented diet, which was the same multiwean feed without phage (Fig. S1 and Table S2). The untreated and treated diets were fed *ad libitum*. Piglets in all groups were dosed with 3 mL of 10% calcium carbonate (Fisher, UK) antacid by oral gavage before introducing fresh feed daily, except on the day of challenge and on the last day of the study.

Piglets in groups T3 and T4 were challenged via oral gavage with a liquid suspension of 10^8^ CFU *S. typhimurium* strain SL1344, and 30 min prior to challenge piglets were dosed with antacid. Piglets in groups T1 and T2 were also dosed with antacid to maintain consistency between the groups. Postchallenge, piglets in all groups were monitored daily via weighing, taking their rectal temperatures, measuring feed intake, and monitoring for depression, respiratory distress and coughing. Fecal samples were also taken daily, and feces was scored based on appearance. Fecal samples were processed on the same day as collection and Salmonella and phage counts were determined. One g of fecal matter was mixed with 9 mL of PBS and vortexed for 3 s, and 0.5 mL was stored at −20°C for the 16S rRNA gene sequencing study. Salmonella counts were determined by spotting 10^−1^ to 10^−8^ dilutions of stool sample on XLD medium agar plates. Plates were incubated overnight at 37°C and colonies were counted. To determine phage counts from samples, the sample was centrifuged and filtered through a 0.22 μm pore-size filter. The filtrate was diluted 10-fold and phage titer calculated from plaque assay on SL1344 after overnight incubation at 37°C (44).

Five days postchallenge, all piglets were humanely euthanized by captive bolt and pithing, followed by exsanguination. Blood samples, tissue, and contents samples from all piglets were taken from the stomach, duodenum, jejunum, colon, cecum, mesenteric lymph nodes, and spleen. Samples were stored on ice and all were processed on the same day as collection. Individually, 1 g of tissue samples, ground by mortar and pestle, and 1 mL of contents samples were mixed with 10 mL of PBS, of which 0.5 mL was frozen at −20°C for the 16S rRNA gene sequencing study. Salmonella and phage counts were determined from all samples using the methods described above. Significant differences in Salmonella and phage counts between treatment groups were determined using *t* tests (Prism 9 version 9.0.2).

### 16S *rRNA* gene sequencing.

On the day of collection, 0.5 mL of fecal and gut compartment samples were stored at −20°C. Microbial DNA from feces and intestinal samples from piglets were extracted using the QIAamp Powerfecal DNA kit (Qiagen, Germany) following the manufacturing instructions. The Qubit was used to quantity DNA samples using the dsDNA HS kit (Thermo Scientific, UK). To construct the 16S rRNA gene library, the procedure outlined by Illumina was followed (https://support.illumina.com/documents/documentation/chemistry_documentation/16s/16s-metagenomic-library-prep-guide-15044223-b.pdf). Three controls were included where 0.5 mL of distilled water was processed through the kit. Following DNA extraction and library preparation, library quantification, normalization, pooling, and sequencing on MiSeq (Illumina, UK) was conducted by Nucleus genomics, University of Leicester, United Kingdom.

Raw reads were processed using USEARCH v11, and the recommended workflow was used as described previously (45, 46) (the workflow is available online too at https://www.drive5.com/usearch/). First, paired reads were merged based on overlapping sequences, and 5091 to 177400 merged reads were identified per sample. Merged reads were stripped of primer-binding sequences and quality filtered to remove low quality reads, and all reads for all samples were combined to generate operational taxonomical units (OTUs). Reads were clustered together based on 97% similarity to generate representative OTUs using USEARCH v11, and OTUs were aligned using the reference database SILVA 16S (47). The relative OTU abundances and taxon were compared between the piglet treatment groups using the Phyloseq software package version 1.26.1 on R (48). ANOVA was used to determine significant changes in relative abundance between treatment groups, and the relative abundance of T1 against T2, T3, or T4 was compared using an unpaired *t* test (Prism 9.0.2). Alpha diversity between the samples was determined using the “estimate richness” function in the Phyloseq package and was calculated using the Shannon and Chao1 diversity indexes. The Mann-Whitney test was used to determine significance in alpha diversity. Beta diversity was determined using the “ordinate: function in Phyloseq, and principal coordinates analysis (PCoA) plots were plotted using the unweighted UniFrac as distance. PERMANOVA analysis was conducted using the Vegan R package using the “adonis” function.

### Patents.

The SPFM Salmonella phages are part of a Leicester patent, pending. Clokie MRJ, Thanki AT. International filing date 09/24/2019. International filing number PCT/GB2019/052695.

### Data availability.

All Sequence files and metadata used in the present study were deposited to the Sequence Read Archive (SRA) under the Bioproject accession PRJNA799037.
