# Supplementary material for: Prophylactic Delivery of a Bacteriophage Cocktail in Feed Significantly Reduces Salmonella Colonization in Pigs
Source: Microbiol Spectr. 2022 May 17;10(3):e00422-22. doi: 10.1128/spectrum.00422-22 (PMC9241700; doi:10.1128/spectrum.00422-22)
Supplement: SUPPLEMENTAL FILE 1 — Supplemental material. Download spectrum.00422-22-s001.pdf, PDF file, 0.8 MB [file spectrum.00422-22-s001.pdf]

Product code: 584 WEANER PELLETS

A complete feedingstuff for feeding to pigs from 8.0kg live weight.

ANALYTICAL CONSTITUENTS

Crude protein	20.00	%	Calcium	0.65	%
Crude fat	5.80	%	Phosphorus	0.60	%
Crude fibre	2.60	%	Magnesium	0.15	%
Crude ash	5.60	%	Sodium	0.31	%
Lysine	1.40	%	Copper (total)	166.0	ppm
Methionine	0.44	%	Selenium (total)	0.38	ppm

COMPOSITION Wheat, Soya (bean) meal feed, dehulled [*], Barley, Cooked Wheat, Soya Beans, extruded[*], Soya Oil[*], Corn, Sugar, Demineralised Whey Powder, Fish Meal, Cooked Barley, Monocalcium Phosphate, Calcium Carbonate, Sodium Chloride, Sodium Carbonate, Sodium Bicarbonate and Onion soluble and grape pip soluble.

ADDITIVES (PER KG)

TECHNOLOGICAL:

E321 BHT 2.4 mg, E563 Sepiolitic clay 148 mg

NUTRITIONAL: 3a672a Vitamin A 10000 IU, E671 Vitamin D3 2000 IU, 3a700 Vitamin E/all-rac-alpha-tocopheryl acetate 75 IU, E1 Iron Sulphate Monohydrate 400 ppm, 3b202 Calcium Iodate Anhydrous 1.6 ppm, E4 Copper Sulphate Pentahydrate 640 ppm, 3b503 Manganese Sulphate Monohydrate 218.8 ppm, 3b603 Zinc Oxide 152.8 ppm, E8 Sodium Selenite 0.6 ppm

ZOOTECHNICAL:

4a1617 Endo-1,4-bèta-xylanase EC 3.2.1.8 1500 EPU, 4a16 6-Phytase EC 3.1.3.26 350 OTU

Contains 125ppm Proviox 50, which contains compounds with anti-oxidant properties to partially replace Vitamin E.

[*]Produced from GM soyabeans. CONTAINS FISHMEAL. DO NOT FEED TO RUMINANTS. For Swine and Poultry: Simultaneous use with water for drinking in which choline chloride has been added should be avoided. Store in a cool dry place. KEEP AWAY FROM CHILDREN. In case of query, please return label.

Supplementay Figure 1. Product label added to pelleted feed with dried phages.

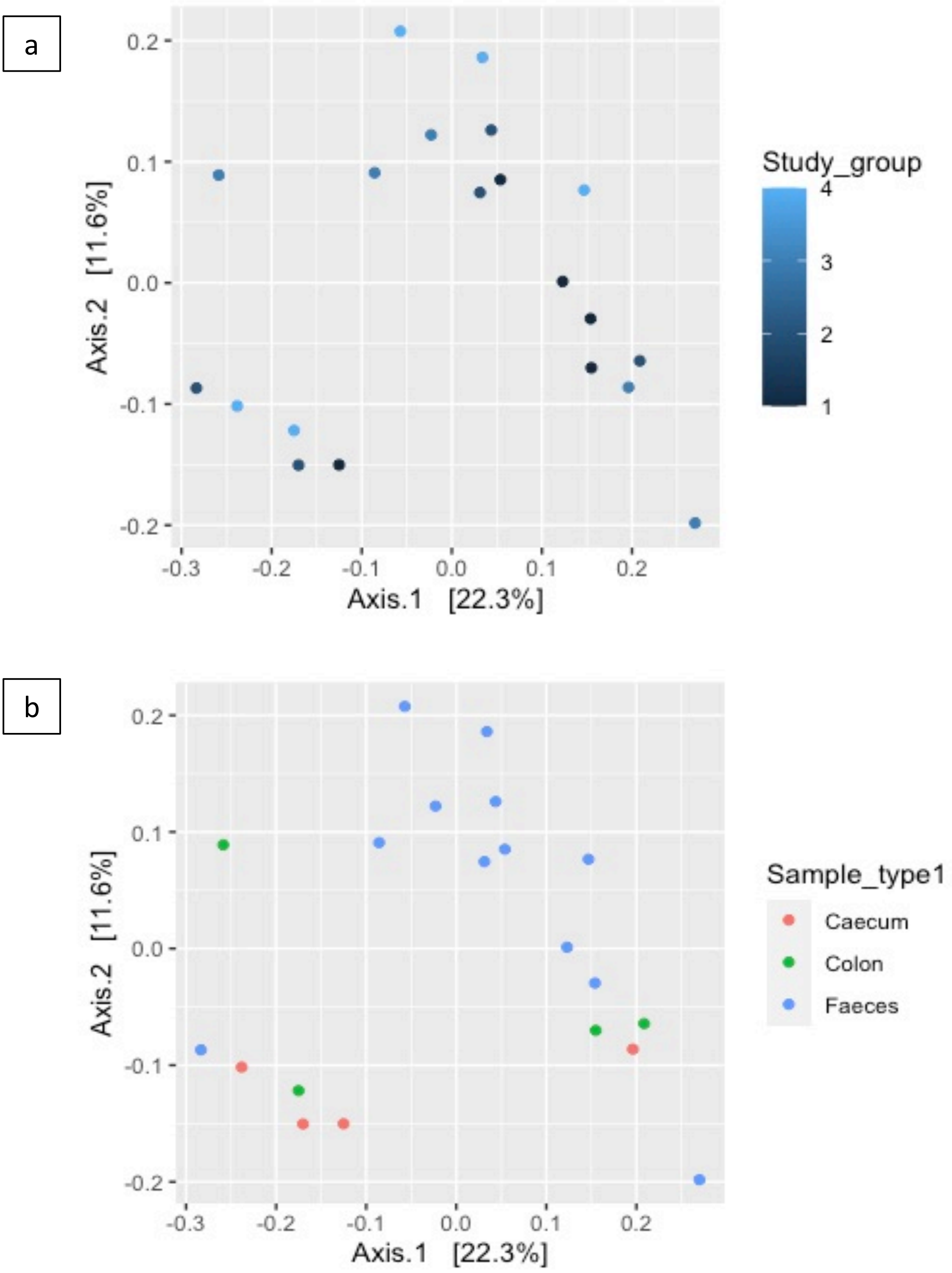

a
Study_group
4
3
2
1
0.2
0.1
0.0
-0.1
-0.2
-0.3
Axis.1 [22.3%]
Axis.2 [11.6%]
b
Sample_type1
Caecum
Colon
Faeces

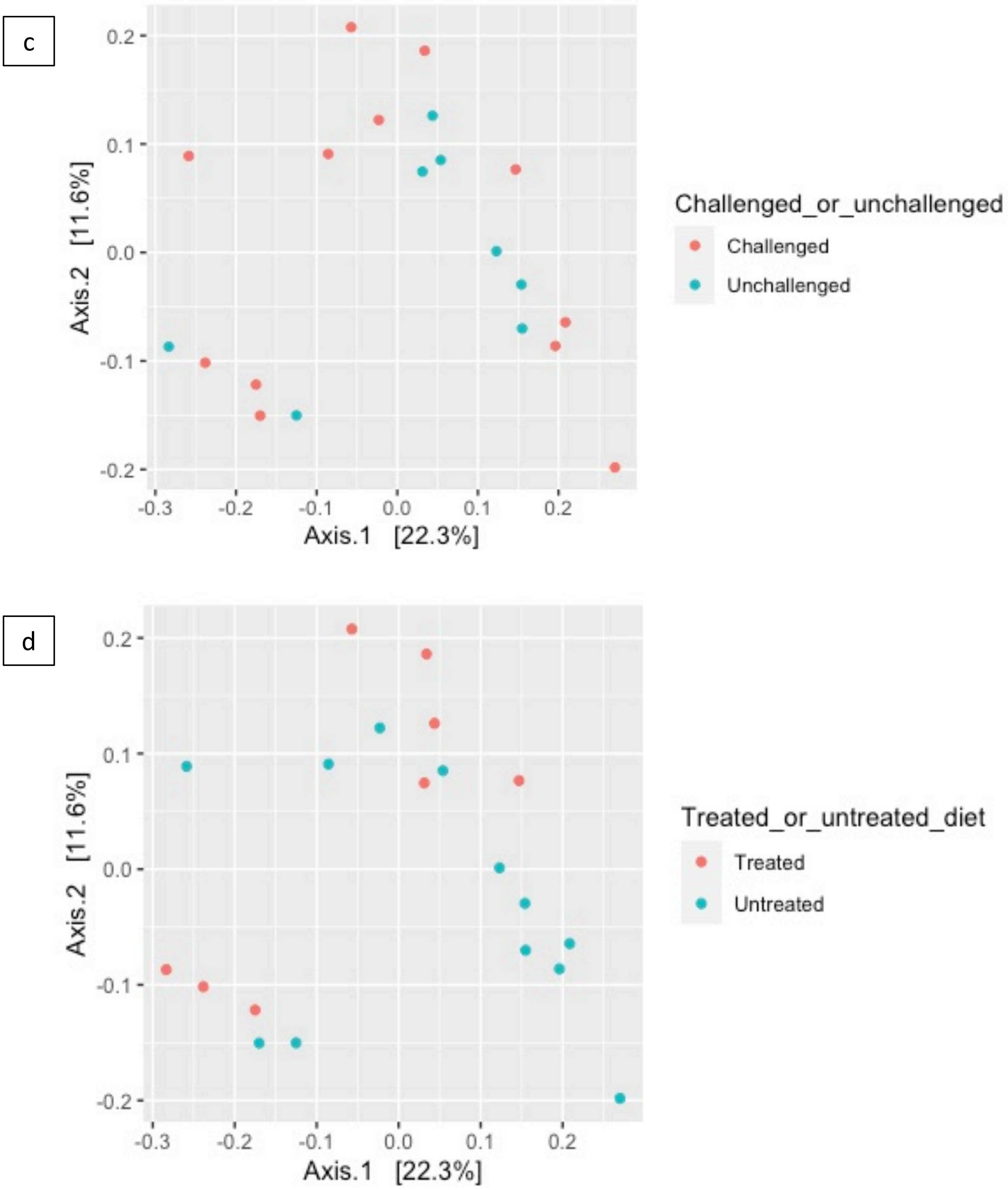

Supplementary Figure 2. Principle coordinates analysis (PCoA) plots using the unweighted UniFrac distance matrix generated from taxon abundances and showing patterns of beta diversity between (a) samples in different study groups (not significant (ns)), (b) different samples types (ns), (c) samples tested from challenged or unchallenged piglets (ns) and (d)

samples taken from pigs fed the treated phage diet or the untreated diet ($p=0.039$). P-value was calculated using PERMANOVA and $p<0.05$ was considered significant.

Supplementary Table 1. The different excipients formulations tested using the commercial spray dryer to minimise phage loss.

Run	Excipient formulation	Final phage titre when mixed with excipients (PFU/ml)	Atomiser[1] (%)	Outlet temperature	Inlet temperature	Moisture content (%)	Average phage recovery (PFU/g)	Drop in phage titre
**A**	8% Trehalose<br>8% mannitol<br>1% leucine<br>1% phage	2.92×10^7	80	70	160	3.00	0	-2.92×10^7
**B**	8% Trehalose<br>8% mannitol<br>1% leucine<br>1% phage	2.92×10^7	80	65	160	3.00	0	-2.92×10^7
**C**	8% Trehalose<br>8% mannitol<br>1% leucine<br>1% phage	2.92×10^7	90	54	125	5.00	9×10^3	-3.24×10^3
**D**	8% Trehalose<br>8% mannitol<br>1% leucine<br>1% phage	2.92×10^7	80	52	120	5.00	1.7×10^4	-1.72×10^3
**E**	8% Trehalose<br>8% mannitol<br>1% leucine<br>1% phage	2.92×10^7	70	52	120	5.12	1.7×10^4	-1.72×10^3
**F**	8% Trehalose<br>8% mannitol<br>1% leucine<br>1% phage	2.92×10^7	60	52	120	5.01	1.2×10^4	-2.43×10^3
**G**	8% Trehalose<br>8% mannitol<br>1% leucine<br>1% phage	2.92×10^7	50	52	120	5.11	1.2×10^4	-2.43×10^3
**H**	8% Trehalose	2.92×10^7	90	52	100	7.23	2.0×10^4	1.46×10^3

	8% mannitol<br>1% leucine<br>1% phage							
**I**	8% Trehalose<br>8% mannitol<br>1% leucine<br>1% phage	2.92×10^{7}	80	52	100	7.10	1.7×10^{4}	1.72×10^{3}
**J**	8% Trehalose<br>8% mannitol<br>1% leucine<br>5% phage	1.46×10^{8}	80	52	100	5.04	1.9×10^{5}	7.69×10^{3}
**K**	8% Trehalose<br>8% mannitol<br>1% leucine<br>10% phage	2.92×10^{8}	80	52	100	5.01	4.6×10^{5}	6.35×10^{2}
**L**	8% Trehalose<br>8% mannitol<br>1% leucine<br>40% phage	1.17×10^{9}	80	52	100	5.03	1.3×10^{5}	8.99×10^{3}
**M**	8% trehalose<br>8% mannitol<br>1% leucine<br>50% phage	1.46×10^{9}	80	52	100	5.39	1.8×10^{5}	8.11×10^{3}
**N**	8% Trehalose<br>8% mannitol<br>1% leucine<br>60% phage	1.75×10^{9}	80	52	100	5.33	5.2×10^{5}	3.37×10^{3}
**O**	16% trehalose<br>8% mannitol<br>1% leucine<br>50% phage	1.46×10^{9}	80	55	100	8.43	2.8×10^{5}	5.21×10^{3}
**P**	16% Trehalose<br>8% mannitol<br>1% leucine<br>60% phage	1.75×10^{9}	80	52	100	5.21	1.1×10^{6}	1.59×10^{3}

**Q**	16% trehalose<br>8% mannitol<br>1% leucine<br>50% phage	1.46×10^9	80	65	110	8.14	8.7×10^3	1.68×10^5
**R**	8% trehalose<br>8% mannitol<br>1% leucine<br>50% phage	1.46×10^9	80	60	100	5.22	1.4×10^4	1.04×10^5
**S**	8% trehalose<br>8% mannitol<br>1% leucine<br>2% euradagit s100<br>50% phage	1.46×10^9	80	60	110	4.56	2.4×10^6	6.08×10^2
**T**	8% trehalose<br>8% mannitol<br>1% leucine<br>2% euradagit s100<br>50% phage	1.46×10^9	80	50	100	5.00	5.3×10^5	2.75×10^3

[1]The atomiser settings refer to a percentage of 50Hz and 100% means the atomiser is rotating at its maximum speed and at 0% its stopped

Supplementary Table 2. Composition of the Provimi early weaner premix added at an inclusion rate of 4.10%.

Description	Premix	Endpoint
Dry matter	98.823 %	4.052 %
Moisture	1.177 %	0.048 %
Crude protein	19.552 %	0.802 %
Crude fat	0.220 %	0.009 %
Crude fat (after acid hydr.)	0.226 %	0.009 %
Crude fibre	0.031 %	0.001 %
Ash	65.716 %	2.694 %
Volume	100 %	4.100 %
Starch (Ewers)	0.215 %	0.009 %
Sugar	0.010 %	0.000 %
Calcium	12.629 %	0.518 %
Phosphorous P	5.051 %	0.207 %
Magnesium	0.322 %	0.013 %
Sodium	6.732 %	0.276 %
Potassium	0.146 %	0.006 %
Chloride	10.148 %	0.416 %
Sulphur, total	1.527 %	0.063 %
P dog. (pigs)	7.274 %	0.298 %
DE pigs	-3.061 MJ/kg	-0.125 MJ/kg
ME pigs	4.838 MJ/kg	0.198 MJ/kg
ME Sch Neu	6.104 MJ/kg	0.250 MJ/kg
NE pigs	3.481 MJ/kg	0.143 MJ/kg
Lysine	9.989 %	0.410 %
Methionine	3.719 %	0.152 %
Meth.+Cyst	3.720 %	0.153 %
Threonine	5.379 %	0.221 %
Tryptophan	0.407 %	0.017 %
Meth./Lysine	0.372 -	0.372 -
Meth.+Cyst./Lysine	0.372 -	0.372 -
Threon./Lysine	0.539 -	0.539 -
Trypt./Lysine	0.041 -	0.041 -
AID Lysine (pigs)	10.271 %	0.421 %
AID Methionine (pigs)	3.807 %	0.156 %
AID Meth.+Cyst (pigs)	3.910 %	0.160 %
AID Threonine (pigs)	5.529 %	0.227 %
AID Tryptohan (pigs)	0.513 %	0.021 %
C18:2 (Linoleic acid)	0.016 %	0.001 %
Fe total	3,659.181 mg	150.026 mg
Fe (sulphate) (E1)	2,926.829 mg	120.000 mg
Cu total	3,913.084 mg	160.437 mg
Cu (sulphate) (E4)	3,902.439 mg	160.000 mg
Zn total	2,740.039 mg	112.342 mg
Zn (oxide) (3b603)	2,682.927 mg	110.000 mg
Mn Total	1,745.685 mg	71.573 mg
Mn (sulphate) (3b503)	1,707.317 mg	70.000 mg
Se total	6.701 mg	0.275 mg
Se (Na-selenite) (E8)	6.098 mg	0.250 mg
Co total	0.165 mg	0.007 mg
I total	24.390 mg	1.000 mg

I (Ca-iodate) (3b202)	24.389 mg	1.000 mg
Mo total	0.000 mg	0.000 mg
Vitamin A (3a672a)	0.244 MIU	0.010 MIU
Vitamin D3 (3a671)	0.049 MIU	0.002 MIU
Vitamin E (3a700)	1,829.268 mg	75.000 mg
Vitamin E eq. (from proviox)	3,048.781	125.000 mg
Vitamin K3 (3a711)	48.780 mg	2.000 mg
Vitamin B1 (3a821)	24.390 mg	1.000 mg
Vitamin B2	121.951 mg	5.000 mg
Vitamin B6 (3a831)	24.390 mg	1.000 mg
Vitamin B12	609.756 mcg	25.000 mcg
Niacinamide (3a315)	731.707 mg	30.000 mg
Pantothenic acid (3a841)	243.902 mg	10.000 mg
Folic acid (3a316)	24.390 mg	1.000 mg
Vitamin C1 monophosphate (3a312)	3,810.732 mg	156.240 mg
Choline chloride	7,317.073 mg	300.000 mg
Acids	7.180 %	0.294 %
ProHacid	9.756 %	0.400 %
6-Phytase (4a16) (Optiphos)	8,536.585 OTU	350.000 OTU
1,4-ß-xyl. (4a1617) (Hostazym X)	36,585.370 EPU	1,500.000 EPU
Cinergy Piglets	9,756.098 mg	400.000 mg
BHT (Butylated Hydroxytoluene) (E321)	58.537 mg	2.400 mg
Xantophylls	0.015 mg	0.001 mg
Flavour/sweetener	9,756.098 mg	400.000 mg
Sepiolite clay (E563)	0.360 %	0.015 %
L-lysine NCl	12.805 %	0.525 %
DL-methionine	3.756 %	0.154 %
L-threonine	5.488%	0.225 %
L-tryptophan	0.415 %	0.017%
Salt	12.537 %	0.514 %
Iron Sulphate Monohydrate	9,756.098 mg	400.000 mg
Copper Sulphate Pentahydrate	15,609.760 mg	640.000 mg
Zinc oxide	3,726.287 mg	152.778 mg
Manganese Sulphate Monohydrate	5,335.366 mg	218.750 mg
Calcium Iodate Anhydrous	38.109 mg	1.562 mg
Sodium Selenite	13.550 mg	0.556 mg
Liquids	0.195 %	0.008%
Density	1.333 T/M3	1.333 T/M3

Supplementary Table 3. Average rectal temperatures of piglets per treatment group.

	**Study Day (°C)**					
**Treatment**	**0 (pre-challenge)**	**1**	**2**	**3**	**4**	**5**
T1	37.8	38.3	38.1	37.6	38.7	38.0
T2	39.1	38.6	38.1	38.9	38.4	38.3
T3	38.9	40.3	38.7	38.4	39.5	38.7
T4	39.2	40.3	39.2	38.7	38.9	38.9

Supplementary Table 4. Average weight of piglets per treatment group.

	**Study Day (kg)**						
**Treatment**	**-8**	**0 (pre-challenge)**	**1**	**2**	**3**	**4**	**5**
T1	7.28	8.10	8.38	8.65	8.91	9.02	9.10
T2	7.33	7.92	8.13	8.48	8.73	9.17	9.40
T3	7.36	7.92	7.83	7.56	7.76	7.89	8.09
T4	7.49	7.94	7.92	7.49	7.41	7.23	7.59

Supplementary Table 5. Average faecal scoring of piglets per treatment group.

	**Study Day (faecal score*)**					
**Treatment**	**0 (pre-challenge)**	**1**	**2**	**3**	**4**	**5**
T1	1.5	1.0	1.0	1.0	3.0	2.5
T2	1.6	1.6	1.3	1.0	1.2	1.4
T3	1.0	1.3	2.0	2.2	2.3	2.7
T4	1.5	2.2	3.5	3.6	3.2	2.9

*Faecal score were categorised as: 1 - Normal, firm but not hard; 2 - Soft, does not hold form; 3 - Runny, spread like pancake mix; 4 - Watery, liquid like orange juice.

Supplementary Table 6. Feed consumed by piglets per treatment group.

	Creep Feed/Untreated Feed Transitional Period					Phage Feed Provision (T2 and T4) and Untreated Feed Provision (T1 and T3)										
Treatment	Feed eaten SD -8	Feed eaten SD -7	Feed eaten SD -6	Feed eaten SD -5	Feed eaten SD -4	Feed eaten SD -3	Feed eaten SD -2	Feed eaten SD -1	Feed eaten SD 0	Feed eaten SD 1	Feed eaten SD 2	Feed eaten SD 3	Feed eaten SD 4	Feed Intake SD -8 to Day -4	Feed Intake SD -3 to Day 5	Total Feed Intake Day -8 to Day 5
	(kg)	(kg)	(kg)	(kg)	(kg)	(kg)	(kg)	(kg)	(kg)	(kg)	(kg)	(kg)	(kg)	(kg)	(kg)	(kg)
T1	0.00	*	0.42	0.34	0.38	0.34	0.34	0.44	0.46	0.44	0.66	0.52	0.40	1.14	3.60	4.74
T2	0.00	*	0.82	0.90	1.00	0.68	0.78	1.10	1.44	1.64	1.94	2.16	2.12	2.72	11.86	14.58
T3	0.00	*	0.76	0.62	0.62	0.90	0.94	1.00	0.46	0.36	0.84	0.86	1.04	2.00	6.40	8.40
T4	0.00	*	0.48	0.70	0.68	0.78	0.78	1.06	0.44	0.22	0.42	0.42	0.60	1.86	4.72	6.58

* Feed contaminated with shavings, amount weighed out greater than amount offered. Amount of feed eaten unknown
